# Editorial: Plant antimicrobial peptides (PAMPs) as biotechnological tools

**DOI:** 10.3389/fmolb.2023.1195043

**Published:** 2023-04-06

**Authors:** Lai Yue Chan, Elizabete Cândido, Sunithi Gunasekera

**Affiliations:** ^1^ Institute for Molecular Bioscience, Australian Research Council Centre of Excellence for Innovations in Peptide and Protein Science, The University of Queensland, Brisbane, QLD, Australia; ^2^ S-Inova Biotech, Programa de Pós-Graduação em Biotecnologia, Universidade Católica Dom Bosco, Campo Grande, Brazil; ^3^ Centro de Análises Proteômicas e Bioquímicas, Programa de Pós-Graduação em Ciências Genômicas e Biotecnologia, Universidade Católica de Brasília, Brasília, Brazil; ^4^ Phamacognosy, Department of Pharmaceutical Biosciences, Biomedical Centre, Uppsala University, Uppsala, Sweden

**Keywords:** antimicrobial resistance (AMR), plant antimicrobial peptide, drug discovery, microbial infections, nanotechnology (drug discovery), agriculture, Biofilm, pharmaceutical

Antimicrobial resistance has been an escalating problem for many years. Diverse living organisms, including plants, animals (e.g., primates, insects, marine sponges), and microbes (e.g., fungi) are important natural resources for the discovery of new antimicrobial peptides, targeting multidrug resistance. Our topic on plant antimicrobial peptides (PAMPs) as biotechnological tools sets a good starting point for bringing together all researchers worldwide in these areas to address this alarming problem. In this topic, we focused on plant immune responses, since it is a vital pathway for a variety of physiological reactions that trigger the production of antimicrobial peptides and metabolites. Many studies are reported in these areas and with incremental drug discovery and development programs for obtaining new antimicrobial drug leads. These studies are not confined to the pharmaceutical industry alone but extend to other areas including the cosmeceutical, agriculture, and veterinary industries.

In this Research Topic, we are delighted to have received three original articles and one mini-review submitted by world-renowned scientists working on antimicrobial research & development, based in Brazil and Mexico. These articles have highlighted the advancements within plant science, providing an excellent overview on the applications of naturally-occurring PAMPs for targeting human diseases and agriculture. The first article is titled “PaDef (Persea americana var. drymifolia), a Plant Antimicrobial Peptide, Triggers Apoptosis, and Induces Global Epigenetic Modifications on Histone 3 in an Acute Lymphoid Leukemia Cell Line” by Paola Jiménez-Alcántar et al. Here the authors obtained evidence that PaDef is a cytotoxic PAMP that is attractive to cell lineages, acting in an antiproliferative manner with an effect associated with chromatin compaction-decompaction promoting gene expression or repression. The **s**econd article is titled “Characterization of *A. nidulans* Biofilm Formation and Structure and Their Inhibition by Pea Defensin *Ps*d2” by Caroline Corrêa-Almeida et al. Here the authors present how the Psd2 defensin has selective activity against fungi, acting directly on the fungal membrane and, in addition, causes no hemolysis of mammalian cells. In short, a new perspective of Psd2 as a promising candidate for the treatment of fungal infections caused by *Aspergillus nidulans* is presented.

The third article titled “Legume Plant Peptides as Sources of Novel Antimicrobial Molecules Against Human Pathogens” by Rui M (Lima et al.). presents small peptide derivatives of Nodule-specific cysteine-rich (NCR) peptides with antibacterial and antifungal activity. The study also provides information about their potential modes of action, highlighting their lack of hemolytic activity as a desirable property for antibacterial drug development. The last article is a mini-review, “Nanoparticles in association with antimicrobial peptides (NanoAMPs) as a promising combination for agriculture development” by Mariana Rocha (Maximiano et al.) Here, the authors indicate nanoparticles as an promising alternative for the delivery of PAMPs for agribusiness applications. The natural and non-toxic attributes of this approach are highlighted as advantageous for society and environment.

Altogether, these studies provide an understanding of AMPs’ biological functions for potential agriculture and pharmaceutical applications. This topic further advocates for the importance of plants as a natural source for discovering new AMPs. To meet the growing challenges associated with bacterial-resistance, these bioprospecting studies looking into new plant sources and yet unexplored peptides are critical in drug discovery efforts. In conclusion, papers published in this Research Topic have made valuable contributions to the field of AMPs, with respect to microbial infections, biochemistry, plant pharmaceuticals, and nanotechnology.

